# Proteomics of *Mycobacterium* Infection: Moving towards a Better Understanding of Pathogen-Driven Immunomodulation

**DOI:** 10.3389/fimmu.2018.00086

**Published:** 2018-01-30

**Authors:** Eik Hoffmann, Arnaud Machelart, Ok-Ryul Song, Priscille Brodin

**Affiliations:** ^1^CNRS, INSERM, CHU Lille, U1019, UMR8204, Centre d’Infection et d’Immunité de Lille (CIIL), Institut Pasteur de Lille, Université de Lille, Lille, France

**Keywords:** host–pathogen interactions, immunometabolism, mass spectrometry, mycobacterial infection, *Mycobacterium tuberculosis*, phagocytosis, phagosome maturation, proteomics

## Abstract

Intracellular bacteria are responsible for many infectious diseases in humans and have developed diverse mechanisms to interfere with host defense pathways. In particular, intracellular vacuoles are an essential niche used by pathogens to alter cellular and organelle functions, which facilitate replication and survival. *Mycobacterium tuberculosis* (Mtb), the pathogen causing tuberculosis in humans, is not only able to modulate its intraphagosomal fate by blocking phagosome maturation but has also evolved strategies to successfully prevent clearance by immune cells and to establish long-term survival in the host. Mass spectrometry (MS)-based proteomics allows the identification and quantitative analysis of complex protein mixtures and is increasingly employed to investigate host–pathogen interactions. Major challenges are limited availability and purity of pathogen-containing compartments as well as the asymmetric ratio in protein abundance when comparing bacterial and host proteins during the infection. Recent advances in purification techniques and MS technology helped to overcome previous difficulties and enable the detailed proteomic characterization of infected host cells and their pathogen-containing vacuoles. Here, we summarize current findings of the proteomic analysis of *Mycobacterium*-infected host cells and highlight progress that has been made to study the protein composition of mycobacterial vacuoles. Current investigations focus on the pathogenicity during Mtb infection, which will allow to better understand pathogen-induced changes and immunomodulation of infected host cells. Consequently, future research in this field will have important implications on host response, pathogen survival, and persistence, induced adaptive immunity and metabolic changes of immune cells promoting the development of novel host-directed therapies in tuberculosis.

## Introduction

Phagocytic cells, such as macrophages, dendritic cells, and neutrophils, represent the first line of defense against invading pathogens, such as *Legionella pneumophila, Coxiella burnetti*, and *Leishmania donovani*, by engulfing and eliminating them by phagocytosis. Phagocytosis is a complex process divided in several steps, which is initiated by the innate recognition of microbial patterns, leading to the formation of microbe-containing vesicles. These vesicles fuse successively with different endocytic compartments [early endosome (EE) and late endosome (LE), and lysosomes (LYSs)] to form a microbicidal organelle, the phagolysosome (1). This process results in the formation of an acidic, oxidative, and degradative environment, which is additionally influenced by immune signals inside and outside of the phagocyte that determine phagosomal fate (2). However, pathogen-containing vacuoles (PCVs) are also occupied and altered by intracellular pathogens providing a successful niche to survive and replicate within phagocytes. Therefore, it is not surprising that numerous microbes developed a wide variety of strategies to adapt and manipulate the phagocytic process (3).

Among these strategies induced by intracellular pathogens, the phagosome maturation arrest initiated by *Mycobacterium tuberculosis* (Mtb) infection allows the colonization of phagocytes and long-term survival within host cells (4). Mtb is causing tuberculosis (TB) in humans with more than 1.3 million deaths each year and an increasing incidence of drug-resistant strains (5). In addition to the lack of an efficient vaccine, tuberculosis remains together with malaria and HIV/AIDS one of the deadliest infectious diseases worldwide. The inter- and intracellular modifications of the host environment induced by Mtb infection are diverse and not fully understood (6). Briefly, after phagocytic uptake, mycobacteria-containing vacuoles (MCVs) are formed, which were shown to interact and exchange material with early endosomal compartments but are devoid of, or only interact transiently, with molecules of LEs and LYSs and are able to retain a pH at around 6.5 within MCVs. The lack of phagosomal acidification is mostly due to a defective retention of the vacuolar proton ATPase (V-ATPase) complex at the phagosome (7, 8). Moreover, it was also considered for long that MCVs are sealed, but recent findings demonstrated that Mtb is able to induce vacuolar rupture, thereby allowing direct interactions of bacterial components with host cytosolic proteins (9–11). Despite numerous studies analyzing the host machinery that controls spatiotemporal vacuolar acidification, progression of phagosome maturation, and intracellular survival of different *Mycobacterium* species, the Mtb-induced delay of phagosome maturation is not fully characterized. Furthermore, we also only begin to understand the impact of the different virulence factor secreted upon Mtb infection, which additionally interfere with host pathways to promote Mtb survival and growth (12). Together with the fact that the existing BCG vaccine is inefficient and needs further improvement and alternatives (13), it is clear that we need more knowledge of mycobacterial pathogenesis and the host factors manipulated by Mtb allowing to establish its intracellular niche. In particular, a better understanding of host immune responses and the involved mechanisms controlling the infection are needed, because they affect disease outcome as well as TB pathology. The possibility of modulating host immunity to maximize mycobacterial killing while minimizing inflammatory tissue damage has received increasing attention in recent years and is applied as novel host-directed therapy against drug-resistant Mtb strains (14).

The development of powerful and sensitive mass spectrometry (MS)-based methods during the past decade allows now the accurate, spatiotemporal identification, quantification, and modification of almost any expressed protein (15). These robust methodologies are increasingly applied to study host–pathogen interactions and to elucidate the proteome of the pathogen itself, of infected cells and of subcellular compartments. In the case of Mtb infection, protein profiling of different mycobacterial strains as well as of clinical, drug-resistant isolates increased tremendously our knowledge about their proteome (16, 17), while MS methods also helped to identify biomarkers of Mtb-infected patients (18). Many previous MS studies on host infection were carried out with non-pathogenic mycobacteria strains or were performed with beads coated with single mycobacterial virulence factors, while data on virulent Mtb strains were scarce. Here, we want to focus on the host side of mycobacterial infection and summarize current findings of the proteomic analysis of *Mycobacterium*-infected cells and the progress that has been made in the purification of PCVs that helped to overcome previous difficulties and will allow the detailed and reproducible proteomic characterization of MCVs to better understand Mtb pathogenicity. In this review, we aim to point out the potential of current MS technology to increase our knowledge of the host response during Mtb infection including pathogen-driven immunomodulation.

## Proteome Analysis of Mycobacteria-Infected Cells

The intracellular niche protects mycobacteria from cellular and humoral component of the immune system. To overcome host cell defense mechanisms, Mtb subverts the normal passage through the endocytic pathway to form a distinct replicative membranous compartment. Furthermore, Mtb is also able to induce vacuolar rupture to reach nutrients in the host cytosol and to escape host defense pathways, both favoring Mtb growth. Therefore, several proteome studies of mycobacteria-infected cells were initiated to collect an inventory of host cell factors required to establish mycobacterial colonization at the cellular level. By using liquid chromatography–tandem MS (LC–MS/MS) approaches for proteome analysis, high identification rates were achieved, which also allow the measurement of the quantitative state of cellular proteomes at any given time of infection (19). In addition, the introduction of stable isotope labeling of proteins prior MS analysis further improved the relative quantification of complex proteomic samples (20). This approach has gained success due to its high proteome coverage, quantification reliability, and high-throughput format. Over the past years, a few MS studies addressed infection settings with virulent and avirulent strains of the Mtb complex, which we have summarized in Table 1A that gave insight on the host environment during mycobacterial colonization.

**Table 1:** List of host proteomic studies of mycobacterial infection performed (A) on total cellular extracts, (B) on isolated cell organelles of infected cells, and (C) specifically on mycobacteria-containing vacuoles (MCVs) and bead-containing phagosomes.

Reference	Type of sample	Experimental design	Peptide labeling	Proteins affected by infection	Main findings/pathways affected by mycobacterial infection
**(A) Mass spectrometry (MS) data of total cellular host proteins**					
Shui et al. (21)	Cellular extract	J774.A1 treated by *Mycobacterium tuberculosis* (Mtb) lipid extract	iTRAQ/SILAC	166	Immune response, oxidation and reduction, signal transduction, vesicle transport, metabolism, etc.
Yu et al. (22)	Lung tissue	Mtb-infected patient tissue, negative for HIV and HBV	Label-free	6 Mtb peptides	Identification of novel Mtb antigens from granuloma lesions, antigen-specific IFN-γ secretion, and functional CTL responses
Kaewseekhao et al. (23)	Cellular extract/supernatant	THP-1 infected by Mtb H37Rv (MOI 1; 1–5 days)	Label-free	283	Cell cycle, antimicrobial and inflammatory responses, DNA replication, etc.
Li et al. (24)	Cellular extract	THP-1 infected by Mtb H37Rv/H37Ra or beads (MOI 35; 12 h)	TMT	235	Apoptosis, blood coagulation, and oxidative phosphorylation
Li et al. (25)	Cellular extract	THP-1 infected by BCG/*Mycobacterium bovis*/Mtb H37Rv (MOI 10; 24 h)	iTRAQ	61	Phagosome maturation and TNF signaling

**(B) MS data of cell organelle proteins**					
Wang et al. (26)	Cytoplasm and nucleus	RAW264.7 infected by *Mycobacterium marinum* (MOI 10; 4 h)	AACT/SILAC	1,429	Toll-like receptor 2-inflammatory responses, major histocompatibility complex-I processing and presentation, and transcriptional factors
Saquib et al. (27)	Endoplasmic reticulum	THP-1 infected by Mtb H37Rv/H37Ra (MOI 10; 12 h)	SILAC	133	Cytosolic Ca^2+^ levels, apoptosis, and cholesterol homeostasis
Diaz et al. (28)	Exosome	THP-1 infected by Mtb H37Rv (MOI 5; 24 h)	Biotinylated	41	Analysis of the exosome content
Kumar et al. (29)	Secretome	THP-1 infected by Mtb H37Rv/H37Ra/BND433/JAL2287 (MOI 10; 6–26 h)	iTRAQ	n/a	Analysis of the secretome
Kuo et al. (30)	Cell and plasma membrane	THP-1 derived DCs treated by heat-killed Mtb powder (10 µg/ml; 3 days)	Deuterium	115	Aminopeptidase N (ANPEP, CD13)
Long et al. (31)	Plasma membrane	THP-1 infected by BCG (MOI 5; 4 h)	SILAC	559	Immune interactions and lipid metabolism

**(C) MS data of MCVs and phagosomes**					
Rao et al. (32)	Phagosome	BMA.A3 infected by Mtb H37Rv/H37Rv ΔfbpA/BCG (MOI 5; 18 h)	Label-free	322	Phagosome maturation and role of the ER in phagosome biogenesis
Lee et al. (33)	Phagosome	THP-1 infected by BCG or beads (MOI 80; 3 h–5 days)	SYPRO Ruby	447	Membrane trafficking and signal transduction
Shui et al. (34)	Phagosome	RAW264.7 incubated with beads coated with ManLAM/PILAM/LPS	iTRAQ	42	Vesicle trafficking, phagosome maturation, and autophagosome
Li et al. (35)	Phagosome	BMDM and BMDC infected by Mtb H37Rv (MOI 5; 18 h)	Label-free	41	Phagosome maturation and antigen presentation
Herweg et al. (36)	Phagosome	RAW264.7 incubated with Mtb or trehalose-dimycolate-coated beads for 30 min	Label-free	835	Mitochondria, cytoskeleton, plasma membrane, ER, endosome, and Golgi-associated proteins

In one of the first studies, Shui et al. published a list of 166 murine macrophage proteins, which showed a differential expression after Mtb lipid exposure (21). Lipids of the mycobacterial cell wall represent approximately 60% of the total bacterial dry weight (37) and were shown to be actively trafficked out of the MCV (38). After both metabolic stable isotope labeling by amino acids in cell culture and chemical isobaric tagging (iTRAQ), the authors found that Mtb lipids act on diverse cellular processes, such as immune response, apoptosis, metabolism, vesicle transport, and signal transduction (21). In addition, also Fc gamma receptor type IIb is upregulated upon Mtb lipid exposure, which is known to block calcium influx and to inhibit phagocytosis and inflammatory responses (39). Moreover, also three proteins implicated in the oxidative burst, which help macrophages to kill intracellular pathogens, were found upregulated upon Mtb lipid exposure: p67phox, p47phox (both subunits of NADPH oxidase), and macrophage migration inhibitory factor. At the cellular level, a recent work compared human THP-1 cells infected by either the BCG vaccine strain or two virulent strains (Mtb H37Rv and *Mycobacterium bovis*) (25). The authors identified 61 host proteins differentially regulated depending on the used infection model with seven of them specifically upregulated upon virulent strain exposure, including CCL20 and ICAM1 involved in TNF signaling. Mtb H37Rv infection also induced upregulation of two interferon-induced transmembrane proteins (Ifitm1 and Ifitm3), which is in agreement with previously published results (40). This study has shown the importance of using appropriate infection models to identify host factors influencing the establishment of a pathogen. In line with this, another recent study identified in human macrophages the downregulation of 235 host proteins, when they compared their profile between macrophages infected with the virulent Mtb H37Rv and the avirulent H37Ra strain (24). The main host cell processes found altered by Mtb infection were blood coagulation, apoptosis, and oxidative phosphorylation. Among these candidates, some proteins showed differential expression levels between both models of infection suggesting the existence of different immunity mechanisms that influence immune responses. For example, the expression of HLA class I, a major histocompatibility complex antigen chain specific to humans, was downregulated after Mtb H37Rv infection, providing evidence that the cross-presentation pathway is affected by Mtb colonization. In another study, Mtb-specific antigens from granulomatous lesions of TB patients could be identified (22), demonstrating the potential of MS-based approaches to study pathogen-driven immune responses.

Although the proteome analysis performed on infected cells and tissue is able to give insight into the host defense mechanisms affected by Mtb colonization, it does not provide specific information about the occupied niche, the MCV, and how its composition and molecular function is altered by the pathogen to allow intracellular growth and replication. Therefore, several labs aim to purify intact cell organelles and PCVs, including those containing different mycobacteria species, to further analyze these compartments by quantitative MS approaches to better understand host–pathogen interactions.

## Current State and Pitfalls of PCV Purification

Traditional organelle enrichment techniques, such as differential centrifugation in (dis-)continuous density gradients and biochemical fractionation, often resulted in preparations of low purity and contained contaminants, such as other cell organelles and molecular constituents. In recent years, additional techniques have been developed, which allow better purification of PCVs enabling organelle proteomics at higher accuracy and reproducibility. The selective enrichment of PCVs is crucial to increase both specificity and quality of sample preparations and can be achieved by immuno-affinity purification, flow cytometry-based single organelle enrichment, and subcellular fractionation in regard of the physicochemical characteristics of the PCVs of interest. Hilbi and coworkers provide a comprehensive overview of different techniques developed to purify vacuoles containing *Legionella, Salmonella, Chlamydia, Simkania*, and a *Mycobacterium* trehalose-dimycolate (TDM) bead model (36). It is essential to control all experimental steps carefully to avoid the presence of contaminating cell organelles and remnants, which will result in the identification of artifacts during MS analysis. In particular, the use of immunomagnetic separation techniques combined with fractionation and density gradient centrifugation has been used successfully to purify vacuoles of intracellular pathogens that allowed the MS-based identification of host proteins recruited to PCVs, such as IRG1, a catalytic enzyme shown to regulate an antimicrobial host response against *Legionella pneumophila in vitro* and *in vivo* (41).

There is a long-standing interest in MCV isolation to identify and characterize pathogen-specific virulence and persistence mechanisms during infection. The first protocols of MCV purification were reported by the Russell laboratory (42, 43) and Pieters laboratory (44) more than 20 years ago and were based on sucrose and/or Ficoll gradient centrifugation. More recently, these approaches have been either modified, e.g., by combining them with iso-osmotic iodixanol density gradients (33), or were replaced by separation techniques, where mycobacteria are magnetically labeled prior infection (45, 46). Currently, the latter methods seem to be superior to purify MCVs since they allow the selective enrichment of pathogen-containing compartments. In all cases, homogenous MCV isolation with a high degree of purity remains technically challenging, especially if one aims to perform it on virulent Mtb strains at BSL3 conditions. However, in combination with methods developed for other intracellular pathogens, for example, the immunomagnetic separation of PCVs using antibodies against species-specific virulence factors (47), will further boost the efforts to unravel the Mtb-specific phagosomal proteome during different times of infection.

## Proteome Analysis of Mycobacteria-Containing Vacuoles

During the last years, several MS studies were published that analyzed different cell organelles of mycobacteria-infected cells (48), such as cytoplasm and nucleus (26), the ER (27), secreted exosomes (28), plasma and cell organelle membranes (30, 31), as well as phagosomes (32–36), which provide detailed insight in the cellular mechanisms of mycobacterial infection. We have listed these findings in Tables 1B,C. For example, in a quantitative MS approach, Kuo et al. performed membrane profiling on human dendritic cells and identified 115 proteins that were upregulated in response to heat-killed Mtb (30). Among those host proteins, aminopeptidase N was found largely overexpressed, and the authors could demonstrate that membranous aminopeptidase N is capable of binding live bacteria and is involved in antigen presentation that impaired T cell activation to facilitate Mtb pathogenesis.

**Figure 1:**
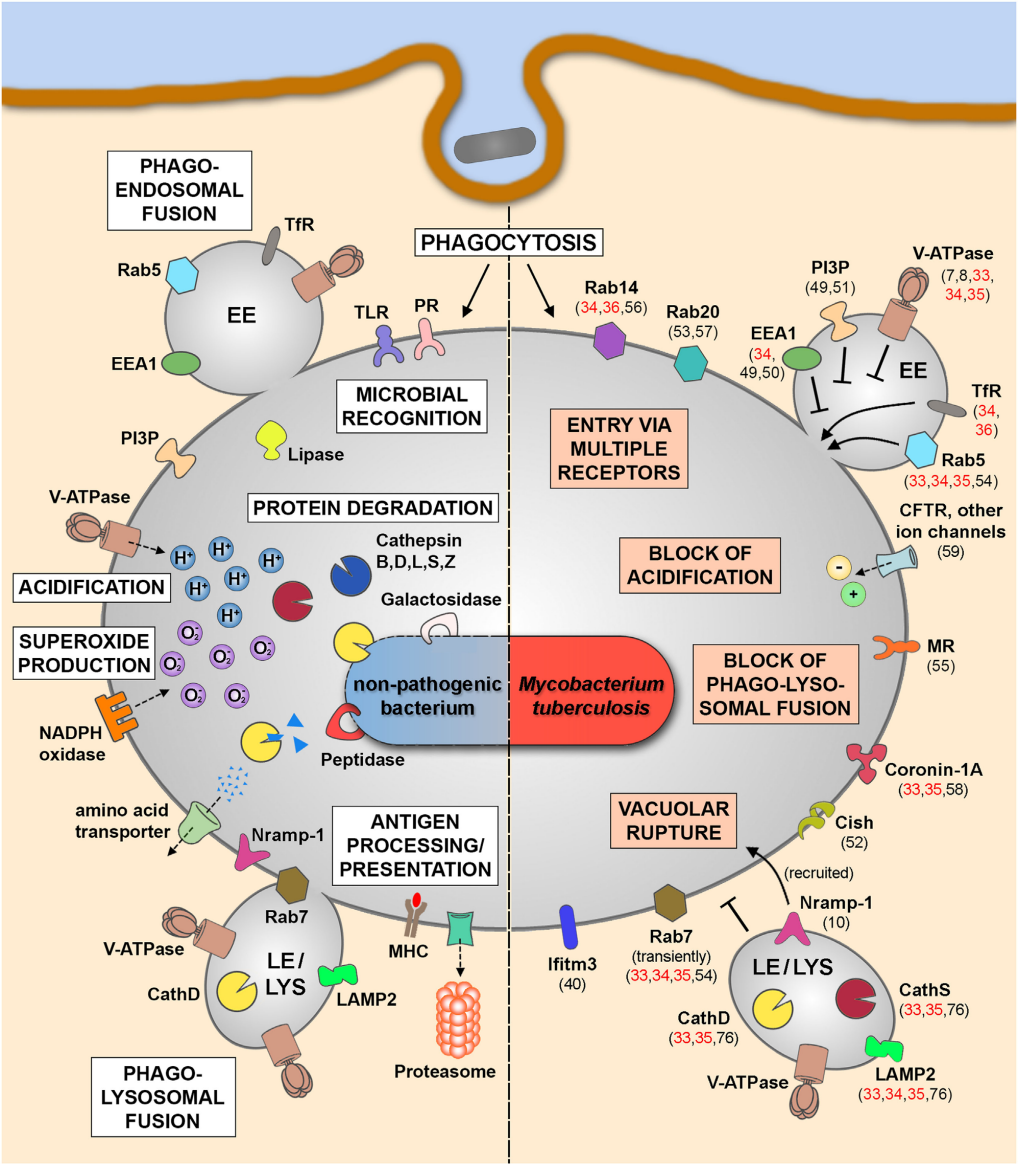
Phagosomal functions after internalization of non-pathogenic bacteria (left panel) and in the context of Mtb infection (right panel). Schematic representation of the key players and main functional features after the uptake of non-pathogenic bacteria leading to the clearance of the internalized cargo (left panel). Upon Mtb infection, the pathogen is internalized into mycobacteria-containing vacuoles (MCVs), which are delayed in phagosome maturation (right panel). Some of the altered phagosomal functions are indicated here together with involved host molecules that were identified by MS approaches (references shown in red) or by non-MS techniques (references shown in black). Abbreviations: CathD, cathepsin D; CathS, cathepsin S; Cish, cytokine-inducible SH2-containing protein; EE, early endosome; EEA1, early endosomal antigen 1; Ifitm3, interferon-induced transmembrane protein 3; LAMP2, lysosome-associated membrane protein 2; LE, late endosome; LYS, lysosome; MHC, major histocompatibility complex; MR, mannose receptor; MS, mass spectrometry; Mtb, *Mycobacterium tuberculosis*; Nramp-1, natural resistance-associated macrophage protein 1; PI3P, phosphatidyl-inositol-3-phosphate; PR, phagocytic receptor; TfR, transferrin receptor; TLR, toll-like receptor; V-ATPase, vacuolar proton ATPase.

The different MS studies on the phagosomal proteome (Table 1C) were performed in immune cells infected by either Mtb H37Rv and *M. bovis* BCG or beads coated with Mtb cell wall components (ManLAM, PILAM, and TDM) and identified phagosomal host proteins that are altered during mycobacterial infection. Together with the findings of non-MS studies investigating MCVs at different time points postinfection, we start to better understand the phagosome maturation block and other features of Mtb infection, which we have illustrated in Figure 1. While molecules such as transferrin receptor and Rab5 (33–36) are commonly found in MCVs, suggesting fusion with EEs, other EE markers are not found or only at much lower quantities, such as phosphatidyl-inositol-3-phosphate and early endosomal antigen 1 (32), confirming previous findings on MCV biogenesis (49–51). The different MS studies (33–35) also confirm the notion that mycobacterial infection actively suppresses the recruitment of the V-ATPase to the MCV membrane (7) to avoid phagosomal acidification due to the secreted Mtb protein tyrosine phosphatase (8). A recent study from us has also shown that the V-ATPase is targeted for ubiquitination and proteasomal degradation during Mtb infection due to the activity of cytokine-inducible SH2-containing protein (Cish) (52). Moreover, Mtb infection also blocks fusion with late endosomal and lysosomal compartments, which excludes molecules such as cathepsin D and S and lysosome-associated membrane protein 2 from MCVs (33–35, 76). Only late endosomal proteins, such as Rab7 and Nramp-1, were shown to be acquired transiently to the MCV membrane (10, 53, 54), the latter being involved in vacuolar rupture and access of Mtb to the cytosol. Recently, the mannose receptor was found to be involved in blocking phagolysosomal fusion by its recruitment of SHP-1 (55). Also two other Rab GTPases, Rab14 and Rab20, were shown to play important roles in MCV biogenesis (34, 53, 56, 57), while other molecules, such as coronin-1A, are actively retained on MCVs containing live Mtb (58). The block of phagosomal acidification, and therefore hydrolase activity, is also supported by the activity of ion channels, such as CFTR (59) and others, which lead to, e.g., high levels of chloride ions in early MCVs (60–62). Finally, there are also other cellular mechanisms, such as autophagy, necrosis, apoptosis, and impairment of antigen presentation, involved in the outcome of mycobacterial infections, which is beyond the scope of this review. However, all these different findings demonstrate an impressive body of evidence, how Mtb interferes with host defense to survive and replicate in different cell types, and how MS analysis of infected cells and of MCVs provide a valuable tool to better understand host–pathogen interactions.

## Conclusion and Outlook

Research on mycobacterial infections includes an increasing number of MS-based approaches, which refine our understanding of the molecular mechanisms underlying Mtb pathogenesis. Together with improvements on purity and reproducibility of sample preparations, in particular of MCVs containing virulent Mtb strains, it is now feasible to combine these methods with other techniques analyzing dynamics and impact of host–pathogen interactions. In addition, current advances in quantitative MS as well as recently emerging targeting MS-based techniques, such as selected reaction monitoring and SWATH MS (17), enable accurate and very sensitive protein measurements. Combined with systems biology-driven workflows, future studies on Mtb infection will allow a better understanding of pathogen-induced changes and immunomodulation of infected host cells.

Future research should focus on the diversity of innate immune cells and their distinct functions that cooperate to control Mtb infection, such as the contribution of myeloid cells and lymphocyte populations (63–65). MS approaches could help to uncover the underlying mechanisms by identifying the involved players. Effective immunity against Mtb requires balanced adaptive immune responses while avoiding damages by immune activation, which determines the persistence of chronic TB infection (66). We now begin to understand the different mechanisms of immune manipulation induced by Mtb infection that favor disease progression, and current interventions aim to reverse these effects by altering the frequency of specific immune cell populations (67). Therefore, novel immunotherapies include depletion strategies targeting regulatory T cells (68, 69) and myeloid-derived suppressor cells (70, 71) to restrict mycobacterial replication. Finally, current findings also demonstrate the importance of metabolic remodeling of immune cells during infection, and how these processes influence effector functions, inflammatory responses, and the outcome of disease (72). How precise changes in metabolites are beneficial for the host during Mtb infection and contribute to protection from TB remains to be uncovered, but the inflammatory state of certain immune cell populations appears to be crucial (73). Without doubt, current MS technology allows the identification of metabolic adaptation during Mtb infection (74) and will allow further insight how immune responses are regulated, for example, the role of different interferons in TB pathogenesis (75). Consequently, future research in these fields will have important implications on host response, pathogen survival, and TB persistence and will promote the development of novel host-directed therapies, particularly against emergent drug-resistant Mtb strains.

## Author Contributions

All the authors participated in the concept, preparation, and writing of the manuscript. EH conceived the content and edited the final version of the manuscript.

## Conflict of Interest Statement

The authors declare that the research was conducted in the absence of any commercial or financial relationships that could be construed as a potential conflict of interest.
